# Meeting Breeding Potential in Organic and Low-Input Dairy Farming

**DOI:** 10.3389/fvets.2020.544149

**Published:** 2020-10-28

**Authors:** Hannah Davis, Sokratis Stergiadis, Eleni Chatzidimitriou, Roy Sanderson, Carlo Leifert, Gillian Butler

**Affiliations:** ^1^School of Natural and Environmental Sciences, Newcastle University, Newcastle upon Tyne, United Kingdom; ^2^Department of Animal Sciences, School of Agriculture, Policy and Development, University of Reading, Reading, United Kingdom; ^3^French Agency for Food Environmental and Occupational Health and Safety (ANSES), Regulated Products Assessment Department, Residues and Food Safety Unit, Maisons-Alfort, France; ^4^Centre for Organics Research, Southern Cross University, Lismore, NSW, Australia

**Keywords:** low-input, organic, dairy farming, milk quality, grazing

## Abstract

Low-input (LI) dairy farming, relying heavily on grazing, is increasing in popularity for perceived sustainability, welfare, and milk nutritional quality benefits. However, there is little research into the breed suitability for these systems. The popular Holstein–Friesians are not well-suited to LI production as, to achieve their potential high yields, they require high levels of concentrate intakes and veterinary inputs. Holstein–Friesians were traditionally bred for high milk yields, which often correlate negatively with functional traits, such as fertility and health. This drives the need for alternative breed choices, and UK dairy farmers use several crossbreeding practices. Additionally, classic measures of production efficiency (kilogram feed per liter of milk) are not the sole priority in LI systems, which also aim for improved health, fertility, forage conversion, and milk quality. This study aimed to explore the effect of breeding strategy on LI and organic production in dairy systems, collecting data from 17 farms throughout England and Wales: 7 organic and 10 low-input conventional systems with both purebred and crossbred cows from different breeds. Records from 1,070 cows were collected, including background data, health, fertility, breeding, and parity. Additionally, milk was analyzed on four occasions (autumn 2011 and winter, spring, and summer 2012). Principal components analysis was used to visualize the effect of management, Farm ID, and stage of lactation on LI production. The analysis clustered cows by Farm ID, showing that individual management practice on each farm had the greatest impact on various production traits. Cows were allocated a composite score based on their yield, health records, and milk fatty acid profile, and a linear mixed-effects model indicated (*p* < 0.01) that crossbred New Zealand Friesian cows scored highest, whereas Dairy Shorthorn cows scored the lowest. This paper highlights weaknesses in current breeding programs for LI and organic farms in the UK, in terms of the alignment of breeds with husbandry practices. Additional research is needed to explore any gene by environment interactions to meet the true potential of individual cows and certain breeds under LI and organic management.

## Introduction

Organic farming in the UK is defined by European Union (EU) regulations (1) and certifying bodies such as The Soil Association (2) and Organic Farmers and Growers (3). However, many farms operate low-input (LI) systems, which are not organic and not formally defined or regulated. LI farming refers to the practice of using fewer inputs than conventional agriculture but not necessarily meeting organic or other quality assurance standards. Motivations toward LI farming include economic, environmental, and social parameters (4). The main criticism of organic and LI farming is that, compared with intensive systems, lower yields require more land to produce the same amount of food, leading to poorer biodiversity if seminatural vegetation is converted to agriculture (5). However, rejecting organic production methods by emphasizing yield productivity ignores opportunities for practices that enhance sustainability; therefore, alternative metrics must assess LI systems. Over the past 60 years, dairy farming has typically focused on making better use of inputs, maximizing profit, relying heavily on high yields and improved feed efficiency (kilogram dry matter intake per liter of milk) [e.g., (6) Milkbench + system]. However, in organic and LI dairying, priorities are different, whereas profit is still essential; the production system involves fewer inputs. Feed efficiency is equally important, but the pathway to achieve this is mainly on reducing external inputs rather than maximizing outputs, a practice that may also benefit herd health (7). Reducing the intensity of production lowers veterinary bills and costs associated with inseminations while using optimal grazing strategies (such as mob-grazing) to enhance soil and sward health, meaning cows consume a richer pasture and produce more nutritious milk (8, 9). A robust method to determine sustainability, accounting for animal health/welfare, nutritional quality, and environmental/social impacts, is needed. Although fully exploring the sustainability of LI dairying is beyond the scope of this study, this paper explores aspects of breeding, production output, health and milk quality of LI, and organic dairy farms.

Traditionally, Holstein/Friesians (HF) has been at the forefront of high yielding dairy production globally. Holsteins are primarily selected for their production traits (milk yield and composition), whereas more traditional Friesians can be selected for functional traits (health and fertility). However, HF cows are not well-suited to LI and organic systems, as they require relatively high levels of both concentrates to achieve maximum yield potential and veterinary inputs (10). Instead, breeds able to maintain health and productivity with LIs are preferred. As a cross, HFs are bred for production traits (higher yield), which are often negatively correlated with functional traits, such as a decline in fertility and health (11). To maximize the potential of both alternative and high-yielding breeds, LI and organic dairy systems have increased their interest in crossbreeding dairy cattle, introducing genetics from more robust breeds (12). Additionally, functional traits are heavily influenced by the local environment and have low heritability (11), making it difficult to select genetic lines to improve health and fertility. For this reason, LI and organic systems benefit from crossing with breeds known to have stronger functional traits.

Organic and LI systems often rely on crossbreeding strategies to optimize their herd health and yield potential. A strong reason for crossbreeding is the resulting heterosis or hybrid vigor in the first generation (F1). Crossbred offspring (including HF crosses) outperformance relative to the parental average is one way to improve functional traits (13) without impacting milk production. However, to extend the benefit beyond the first generation, a carefully designed system is required for rotational crossbreeding: crossing two F1 individuals only expresses half the hybrid vigor, whereas introducing a third breed preserves up to 86% of the heterosis (12). Crossbreeding high-production HF with traditional breeds better suited to LI management (with high forage diets) shows potential. For example, recent studies comparing breeds and crossbreeding regimes in Switzerland and the UK showed more traditional breeds, or crossbreeding with traditional breeds can significantly improve the economic performance and milk quality in LI grazing based dairy systems (10, 14). The indicators from these studies are positive, but further research is needed to identify the key mechanisms required to produce predictable, repeatable, efficient, and effective crossbreeds.

There is very little recognized research into breeding for crossbred cattle in smaller LI and organic dairy systems. Yet, these farms have progressed with crossbreeding for many generations within their herds, each using a different strategy to search for breed combinations that perform within their system (15). Therefore, there is not a clear breed (or crossbreed) that typically outperforms others in LI systems, in the way that HF dominates conventional production. In addition, most scientific research has focused on HF because they account for 95% of the EU dairy cow population (16). UK organic milk was valued at £351 million in 2018, with over 25% of UK households purchasing organic milk, representing 5.1% of retail milk sales (17), highlighting the increased need to develop appropriate crossbreeding schemes for such production chains. Studies from a range of countries argue that, due to genetic × environment (GxE) interactions, optimal genetic progress requires either independent breeding programs or an index (to rank sires against requirement) specific for each farming system (18–23). This approach would directly benefit LI and organic systems.

The complexity of breeding support for LI dairying is not well-established in the UK. In LI and organic dairy systems, the diet is predominantly forage; therefore, it is beneficial to have cows that efficiently convert forage, especially grazing, to milk (24). However, current UK breeding objectives available do not include forage conversion as a desirable trait when calculating economic values of genetic gain. Instead, the Agriculture and Horticulture Development Board (AHDB-UK levy board funded by farmers and growers) breeding index for year-round calving focuses on milk production (34.4%), health (21.8%), fertility (15.3%), and temperament, among other traits (25). The AHDB also has a Spring Calving Index, aimed at herds making use of grazed grass by assigning 71.6% of the weighting to fitness traits, but the dominant individual driver is still production (27.4%), and the link between efficiency (with an emphasis on forage conversion) in LI systems has yet to be fully explored. Typically, LI and organic management that supports animal health and mastitis is the main concern (26), whereas health and fertility remain essential in these systems; the risk of illness (for example, acidosis) is much reduced. Although these UK resources for dairy breeding selection exist, other options seem more appropriate for organic and LI production.

Milk quality has gained a lot of media attention recently, continuing the debate around the role of milk in human diets and the environment (27). Milk fatty acid (FA) profile is strongly influenced by management, and there is a clear difference in the FA profiles of organic and conventional milk (28–30) between the different stages of lactation (31) and seasonally (32, 33). Additionally, FAs can vary as much within- as between-breeds (34, 35), making it harder to isolate breeds that could give an “optimal” FA profile within a specified management system. Some FAs have been studied closely for their effects on human health. The main FAs considered to have a positive effect on human health are alpha-linolenic acid, eicosapentaenoic acid (EPA), docosapentaenoic acid (DPA), docosahexaenoic acid (DHA), oleic acid (OA), and cis-9 trans-11 conjugated linoleic acid (CLA9). Alpha-linolenic acid is the most abundant omega-3 (n-3) FA and promotes healthy aging and fetal development (36, 37). The long chain n-3 FAs, EPA, DPA, and DHA are anti-inflammatory and reduce the risk of coronary heart disease (CHD) (38). OA can reduce the risk of CHD and promotes stable cellular membranes (39). CLA9 has been shown to lower the risk of CHD and enhance the immune system (40, 41). In contrast, FAs highlighted as undesirable in human nutrition due to their association with increased CHD risk are lauric (C12:0), myristic (C14:0), and, in particular, palmitic (C16:0) acids (39). Also, the most abundant omega-6 (n-6), linoleic acid, is an essential FA in human diets, but if total n-6 is in excess, as prevalent in Western diets, it becomes pro-inflammatory with negative health effects (42). Of greater relevance is the dietary ratio of n-6/n-3, which, when too high (the exact optimal ratio is unknown), may cause inflammations and increase CHD risk (42, 43). Although there is currently no premium in UK linked to milk fat composition, in the USA, CROPP's organic “Grassmilk™” receives a 15% premium above standard organic milk prices for meeting minimum requirements for the n-6/n-3 ratio, total n-3, and CLA (29). This demonstrates the potential for other sectors and countries to create premium dairy products with an increased concentration of beneficial FAs.

Historic approaches to breeding in dairying have not taken a whole system view, generally resulting in poor health traits and concentrate-dependent cows (16). If robust methods to identify cattle that best suit a particular system are to be developed, there is the potential to improve animal health and welfare, production, nutritional quality, milk FA profile, and efficiency. This paper aims to identify breeds within LI and organic dairy systems that can maintain health and yield while producing milk with a beneficial FA profile. The objectives are to (a) define the variables most relevant to LI and organic farming and observe differences in the management system (individual farms), (b) identify breeds that are similar across the farms and quantify differences, (c) develop a score for LI-production (LI-P) to identify breeds that best suit LI and organic production in terms of production, health, and milk composition with respect to consumer health.

## Materials and Methods

### Data Collection

Data for this study were collected from 17 dairy farms (7 organic and 10 LI-conventional) throughout England and Wales between November 2011 and October 2012. All herds were a mix of both purebred and different crossbred cows (Table 1). Herd sizes ranged from 150 to 550 cows, and a total of 1,070 cows were recorded to encompass a broad range of breeds and crosses from each farm. A one-off questionnaire was completed to gain information on pre-survey health and parity as well as a breeding pedigree for all individual cows (according to the farmers' records). Milk from each cow was sampled over four dates: autumn 2011 (D1), spring (D2), summer (D3), and autumn 2012 (D4). A corresponding questionnaire for each farm and cow was used to record husbandry practices on all sampling dates, including milk yield, disease incidence, health treatments, cow diet, calving intervals, milking, and grazing management. Organic farming standards require concentrate feed to be sourced organically and have strict land management application practices (2), whereas LI follow similar practices but are not certified organic. Organic and LI farms fed similar levels of concentrate per cow, and organic farms typically fed more conserved forage (Supplementary Table 1). Access to grazing varied across the year and individual management (Supplementary Table 2). All milk samples were analyzed for basic composition, somatic cell count (SCC), and FA profile. All procedures were acceptable to internal ethical review, in accordance with EU Directive 2010/63/EU for animal experiments and approved by the Animal Welfare and Ethical Review Body at Newcastle University.

**Table 1:** Background information on each farm.

**Farm** **ID**	**Management**	**No. of cows included**	**Calving**	**Breeds and crosses** **represented^a^**
1	Organic	40	Spring	AYR, JE, HF, NZF, SR, SH
2	Organic	42	Year-round	HF, JE, SR
3	Low-input	55	Spring	BS, JE, HF, SR
4	Low-input	52	Spring	NZF, JE, HF
5	Organic	49	Year-round	HF, SR, SH, MRI
6	Low-input	28	Spring	HF, JE, SR
7	Organic	61	Autumn (late)	AYR, HF, SH, SR
8	Low-input	113	Year-round	BF, HF, SR, SH, MRI
9	Low-input	60	Autumn (early)	BF, JE, HF, NZF, SH
10	Organic	55	Autumn (early)	BF, BS, HF, MO, SR
11	Low-input	66	Spring	JE, NZF, BF, HF
12	Low-input	27	Spring	BF, SR, JE
13	Low-input	84	Year-round	AYR, BF, HF, SR, MO, NZF
14	Low-input	76	Spring	BF, JE, NZF, SR, HF, MRI
15	Organic	93	Autumn	AYR, HF, MO, SR, JE
16	Low-input	97	Spring	AYR, JE, HF, NZF
17	Organic	72	Autumn	AYR, HF, SH, XX

a *HF, Holstein/Friesian; NZF, New Zealand Friesian; BF, British and unknown Friesian; JE, Jersey; SR, Scandinavian Red; SH, Shorthorn; AY, Ayrshire; MO, Montbelliarde; BS, Brown Swiss; MRI, Meuse Rhine Issel; XX, crossbred with unknown breed composition*.

### Milk Analysis

A representative raw milk sample was collected from each cow during milking in the parlor on each sampling date. Milk samples were preserved with Bronopol and kept at ambient temperature during transportation to a commercial National Milk Recording (44) lab. Basic milk composition was analyzed using Milkoscan FT 6000 (Foss Electric, Hillerød, Denmark) (milk fat, protein, urea, and lactose content), and SCC was recorded using a Fossomatic instrument (Foss Electric). The samples were then transported at ambient temperature (10–25°C) to Newcastle University, frozen at −20°C. Bronopol preserves milk for more than 5 days and is effective unless temperatures are consistently high (45); ambient temperature varied by season, but milk was frozen within 4 days of collection. There is some evidence that Bronopol may have a small impact on minor long-chain FAs (46) and protein concentration (47). However, all milk samples in this study were treated the same and are therefore comparable. Milk was defrosted at 4°C, stirred thoroughly to homogenize, and 3–4 ml of milk was transferred in a 7-ml container, frozen at −20°C, and freeze-dried. The lipid was extracted using the method described by Chilliard et al. (48), where 130 μg of lyophilized milk was methylated and esterified. Gas chromatography (Shimadzu, GC-2014, Kyoto, Japan) equipped with a flame ionization detector and by using a Varian CP-SIL 88 fused silica capillary column (100 m × 0.25 mm ID, 0.2 μm film thickness) was used to analyze the FAs. The gas chromatography method has been previously described by Stergiadis et al. (49). Individual FAs were identified against peaks generated by a 52 methyl FA standard, with the area under each peak quantifying the relative proportion of each in the total FAs. An FA methyl ester standard and published chromatograms (50, 51) were used to identify the FAs, and correction factors for short-chain FAs were applied using the method described by Stergiadis et al. (49).

### Data Handling

#### Breed Combinations

The farmers' breeding records categorized all animals. Cows were given a code based on their sire, dam, and predominant breed, for example, a pure-bred Jersey = JE; sire Jersey and dam Ayrshire = JEAYR; sire Jersey × Shorthorn and dam Jersey × Ayrshire = JEX (Table 1). The X indicates a majority genetic contribution and/or a back cross. Including the sire and dam breeds for all cows across the study resulted in around 40 different breed combinations of varying population sizes, depending on the sampling date. This ranged from a single representative on one farm (British Friesian × Montbelliarde) to 119 HF individuals across all farms for D2. To rationalize the number of crossbreed combinations in this study, there is no differentiation between the contribution of genetics by parents' sex. For example, both a cross from a Jersey sire and HF dam and from an HF sire and Jersey dam are labeled HFJE.

#### Data Analysis

Microsoft Excel was used for data handling, whereas all statistical analysis was completed using “R” (52). The background information on the farms and monitored cows is displayed in Table 1.

#### Low-Input-Production and Principal Components Analysis

The initial data collection involved 1,070 cows, but for some farms and/or cows on some sampling dates, there are missing and incomplete records. For the observational statistics, the cows selected had records on any given date for production, health, and FA composition results (explained later). This resulted in 299, 757, 772, and 613 cows on D1, D2, D3, and D4, respectively.

Focusing on the available data, using a combination of farm records and results from milk analysis and the priorities of typical LI practices, the variables selected to define LI-P were split into three main criteria:

1. Production:
  - Milk yield (L/day).
  - Total fat and protein solids (kg/day).
2. Health:
  - Udder health; SCC (×10^3^ cells/ml milk).
  - Treatments, including antibiotics (e.g., for mastitis or metritis) or other (e.g., for lameness, milk fever, or pain/inflammation).
3. Fatty acid profile:
  - Percentage of total profile with desirable FAs (n-3, OA, CLA9, EPA+ DPA+ DHA).
  - Percentage of total profile with FAs often consumed in excess and undesirable (C12:0, C14:0, C16:0, n-6, and n-6/n-3 ratio).

**Figure 1:**
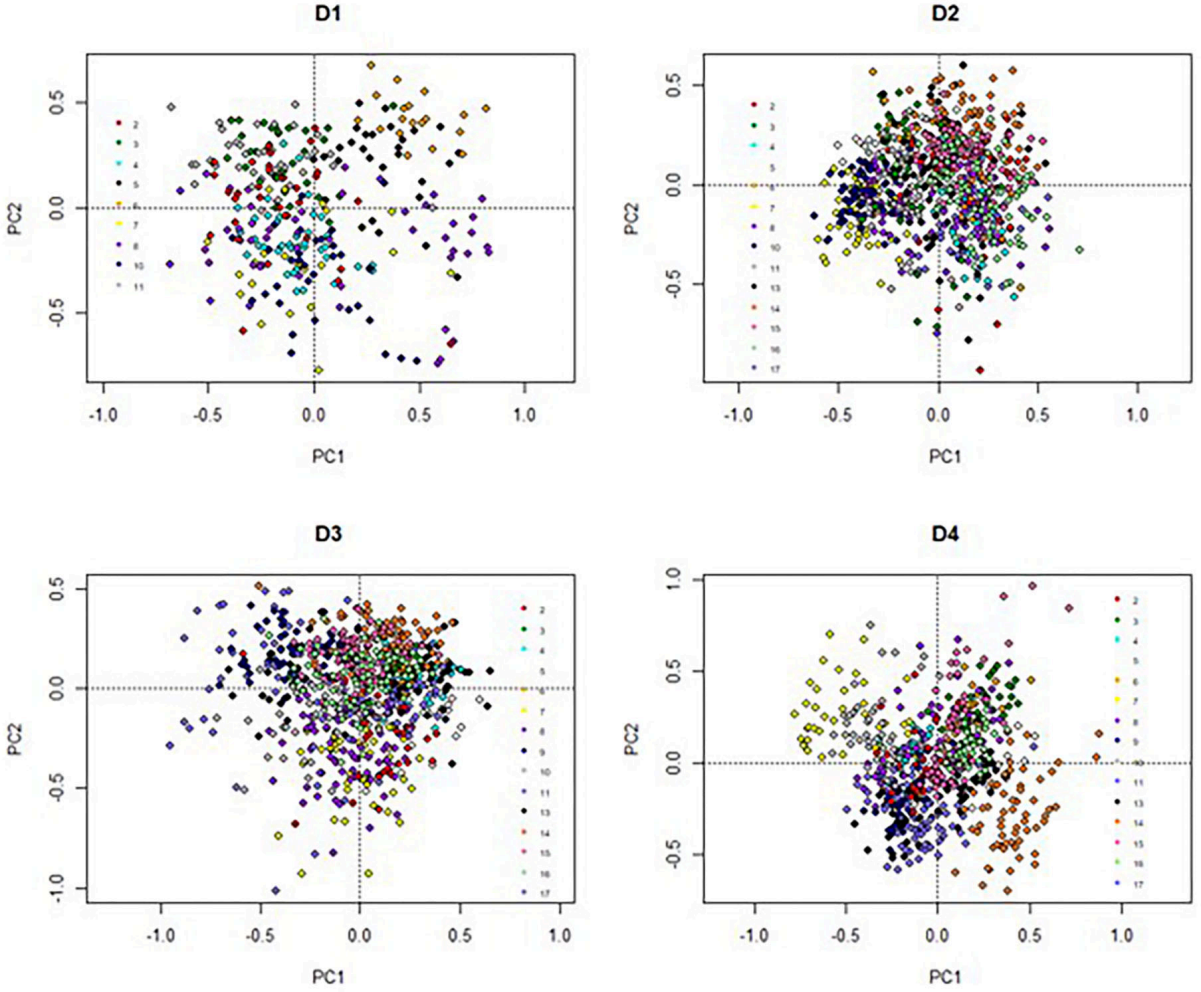
Principal components analysis based on low-input-production, highlighted by Farm ID. C12:0 = Lauric Acid, C14:0 = Myristic Acid, C16:0 = Palmitic Acid, CLA.9 = Conjugated linoleic acid (C18:2, c9t11 isomer), n3 = omega-3, n6 = omega-6, n6n3 = omega-6/ omega-3 ratio, EPA + DPA + DHA = EPA = Eicosapentaenoic Acid + DPA = Docosapentaenoic Acid + DHA = Docosahexaenoic Acid, SCC = Somatic Cell Count, Treatments = Health Treatments.

**Figure 2:**
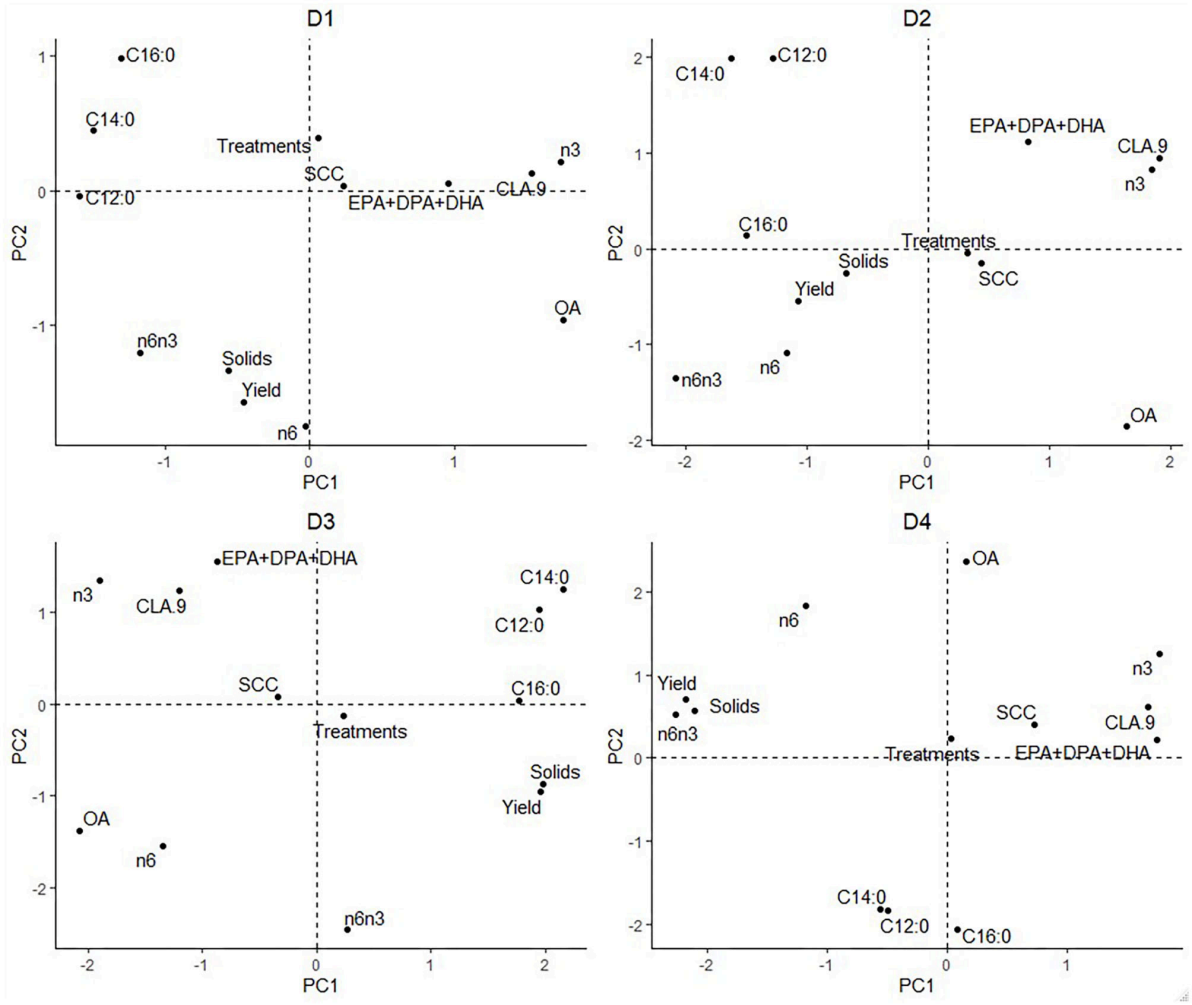
Principal components analysis displaying characteristics of low-input-production.

The elements of LI-P had different units (FAs were proportional, yield: liters/cow/day, SCC: × 10^3^ cells/ml milk, etc.); thus, the data was standardized (normalization to mean of zero and standard deviation of one) (53) to give each element of LI-P the same weight. Principal components analysis (PCA) in the package “vegan” (54) was used to aid visualization of the effects of Farm ID (2–17) on LI-P. Two sets of graphs were produced from the PCA. First, graphs (Figure 1) in which points represent samples/records from cows (at each farm, one graph for each the four dates), where the closer two points are to each other in PCA ordination space, the more similar their characteristics (in terms of production, health, and milk FA profile). The points in these graphs were color-coded by farm identity to aid interpretation. Second, PCA graphs of these characteristics (Figure 2), in which points close together, indicate co-occurrence on similar farms or farming systems. This second set of PCA graphs were also broken down by date. In other words, the characteristics that are grouped together in Figure 2 can be associated with cows and/or farms that occupy similar ordination space in Figure 1.

#### Descriptive Statistical Analysis

For the descriptive statistical analysis, additional inclusion criteria were considered: on any sampling date, records existing for at least six cows of the same breed (combination) from at least three different farms. These criteria resulted in the most breed combinations and ensured comparison between breeds rather than individual farm management style. After these additional inclusion criteria had been applied, there were eight breeds for comparison: Ayrshire cross (AYRX, *n* = 100), HF (HF, *n* = 325), HF × Jersey (HFJE, *n* = 184), HF × Scandinavian Red (HFSR, *n* = 274), Jersey cross (JEX, *n* = 121), New Zealand Friesian cross (NZFX, *n* = 90), Dairy Shorthorn (SH, *n* = 80), and Scandinavian Red cross (SRX, *n* = 140). The number of cows represented by each breed from each farm is available in Supplementary Table 3.

The “R” package “nlme” (55) was used to model “Breed” against the variables described for the LI-P, with Season and Farm ID as random factors. The linear mixed-effects model accounts for variation explained by the fixed effects (Breed) and random effects (Season and Farm ID). As farms were observed across the four sampling dates, these related measures would violate the independence assumptions made by a linear model, hence, the use of “Farm ID” and “Season” as random factors. Days in milk did not differ between the breeds (F-statistic = 1.50, *p* = 0.165), allowing the breed to be compared without differentiating or adjusting for the stage of lactation. On each date all cows from the same farm were fed the same ration, not as individuals (Supplementary Table 1). The feed data did not meet the assumptions of the model; therefore, mean and standard deviations are given, but a *p*-value is not provided. Observationally, there was no big difference in the amount of concentrate fed between the breeds, but there was a notable difference in the amount of conserved forage-fed between breeds (Table 2). Concentrate feeding is thought to have the biggest impact on the FA profile (56); therefore, no corrections were made to the data before analysis.

**Table 2:** Effect of breed on components of low-input-production: production (milk yield and total fat and protein solids), health (health treatments and SCC), and nutritionally relevant FA in milk (expressed as a percentage of the entire FA profile).

	**AYRX^a^**	**HF**	**HFJE**	**HFSR**	**JEX**	**NZFX**	**SH**	**SRX**	**Sig^b^**
*n*	100	325	184	274	121	90	80	140	
Days in milk	154 ± 94.25	182 ± 96.89	157 ± 102.23	161 ± 94.26	134 ± 85.21	138 ± 96.88	153 ± 106.91	151 ± 93.30	ns
Concentrate feed (kg/day)	3.0 ± 1.54	3.3 ± 2.15	3.6 ± 1.68	4.5 ± 2.45	3.1 ± 2.49	3.5 ± 3.00	3.3 ± 1.49	3.1 ± 2.55	NA
Conserved forage (kg/day)	3.7 ± 4.91	6.1 ± 5.25	6.5 ± 5.65	5.6 ± 5.48	3.1 ± 3.80	1.7 ± 2.94	8.2 ± 4.78	3.2 ± 4.09	NA
**Production**									
Yield (L/day)	20.2 ± 7.33	21.2 ± 8.67	21.8 ± 8.89	21.9 ± 9.50	17.9 ± 7.47	20.1 ± 7.08	17.8 ± 8.81	19.7 ± 7.68	***
Solids (fat and protein) (kg/day)	1.7 ± 0.799	1.6 ± 0.618	1.8 ± 0.704	1.7 ± 0.627	1.7 ± 0.652	1.7 ± 0.555	1.5 ± 0.763	1.6 ± 0.516	***
**Health**									
SCC (×10^3^ cells/ml milk)	243 ± 454.2	234 ± 584.5	248 ± 782.1	293 ± 885.4	247 ± 664.1	232 ± 944.9	261 ± 688.2	170 ± 400.8	ns
Treatments	0.41 ± 0.818	0.34 ± 0.713	0.35 ± 0.670	0.24 ± 0.549	0.12 ± 0.369	0.08 ± 0.343	0.09 ± 0.284	0.18 ± 0.527	**
Median SCC (×10^3^ cells/ml milk)	78.5	78.0	70.0	73.0	84.0	56.5	89.0	73.0	
**FA profile**									
C12:0^c^	3.2 ± 0.797	3.3 ± 0.807	4.0 ± 0.918	3.6 ± 1.003	3.9 ± 0.832	3.4 ± 0.822	3.7 ± 0.844	3.8 ± 0.771	**
C14:0	10.9 ± 1.82	11.4 ± 1.53	12.2 ± 1.69	11.8 ± 1.56	11.6 ± 1.45	10.7 ± 1.74	11.6 ± 1.49	11.9 ± 1.40	**
C16:0	29.6 ± 4.79	32.5 ± 4.92	32.9 ± 6.23	31.4 ± 3.99	31.4 ± 6.32	29.6 ± 5.10	29.9 ± 3.93	31.3 ± 5.68	***
n-6	1.6 ± 0.300	1.7 ± 0.532	1.6 ± 0.419	1.6 ± 0.462	1.4 ± 0.434	1.6 ± 0.437	2.1 ± 0.454	1.4 ± 0.444	**
n-6/n-3	1.0 ± 0.309	1.4 ± 0.655	1.4 ± 0.441	1.3 ± 0.485	1.1 ± 0.526	1.3 ± 0.765	1.9 ± 0.887	1.1 ± 0.514	*
OA	20.3 ± 3.87	18.8 ± 3.87	16.6 ± 4.02	19.5 ± 4.01	17.8 ± 4.45	20.2 ± 4.22	20.2 ± 3.46	18.6 ± 4.20	**
CLA9	0.99 ± 0.418	0.88 ± 0.507	0.67 ± 0.451	0.79 ± 0.416	0.93 ± 0.491	1.03 ± 0.454	0.74 ± 0.602	0.91 ± 0.417	ns
EPA + DPA + DHA	0.23 ± 0.074	0.20 ± 0.073	0.19 ± 0.056	0.19 ± 0.046	0.23 ± 0.070	0.22 ± 0.085	0.20 ± 0.083	0.21 ± 0.056	**
n-3	1.7 ± 0.460	1.4 ± 0.513	1.3 ± 0.450	1.3 ± 0.295	1.4 ± 0.412	1.5 ± 0.504	1.4 ± 0.650	1.4 ± 0.295	***

a *AYRX, Ayrshire cross; HF, Holstein/Friesian; HFJE, Holstein/Friesian × Jersey; JEX, Jersey cross; NZFX, New Zealand Friesian cross; SH, Shorthorn; SRX, Scandinavian Red cross*.

b P-values < 0.05. ***P < 0.001, **P < 0.01; *P < 0.05, t: P < 0.1, ns: P > 0.1.

c *C12:0, Lauric Acid; C14:0, Myristic Acid; C16:0, Palmitic Acid; n-6, omega-6; n-6/n-3, omega-6/omega-3 ratio; OA, Oleic Acid; CLA9, Conjugated linoleic acid (C18:2, c9t11 isomer); EPA + DPA + DHA, eicosapentaenoic acid + docosapentaenoic acid + docosahexaenoic Acid; n-3, omega-3*.

*Mean ± standard deviation (SD) and ANOVA p-values*.

Traditionally, *post-hoc* Tukey honest significant difference tests are used for multiple comparisons of levels within a factor. However, due to the complexity of this data set with multiple levels of comparison (8), some with few replicates, controlling the familywise error rate even by this approach would risk numerous type 1 errors (false-positives) and would be misleading (57–59).

#### Low-Input Production Score

To create a universal score for each record, common units are required. Using the variables selected for LI-P, scores were created for each cow record to assess the best performing breed. Milk yield, total fat and protein solids, SCC, and proportions of desirable and undesirable FAs were (higher rankings indicate more beneficial qualities) scored as described next.

1. Production records [milk yield (L/day) and total fat and protein solids (kg)] were allocated into five groups of equal observations, rated 1–5 with 5 the highest and 1 the lowest. Scores were combined to make a total production score, out of 10.
2. SCC (×10^3^ cells/ml milk) was allocated into five groups of equal observations rated 1–5 with 5 the lowest and 1 the highest. For veterinary treatments, cows were given a 1 if they received no treatments and 0 if they had been given antibiotics or an alternative (e.g., for mastitis or metritis or other, e.g., for lameness, milk fever or pain/inflammation) at least once since the previous collection date, which was added to the SCC category resulting in a total health score, out of 6.
3. For desirable FAs (OA, CLA9, n-3, and EPA + DPA + DHA), concentrations were ranked and allocated to five equal groups with a score of 5 was given to the highest and 1 to the lowest group, whereas undesirable FA (often consumed in excess) (C12:0, C14:0, C16:0, n-6, and n-6/n-3 ratio) scores were reversed, 5 to the lowest group. FA categories were combined to create an FA score, out of 45.

These individual assessments were then used to calculate a single score (out of one) for each cow record using two alternative approaches. The score weightings are based organic and LI values, the AHDB Spring and Autumn calving indices (60) and the premium offered for FA quality by Organic Valley's Grassmilk® (29).

- Weighted health score: the scores were weighted at 30% production, 50% health, and 20% FA.
- Weighted production score: 60% production, 30% health, and 10% FA.

For example:

Weighted health score = 30% ^*^ (production score/10) + 50% ^*^ (health score/ 6) + 20% ^*^ (FA score/45).

## Results

### Low-Input-Production and Principal Components Analysis

The PCA result is displayed in Figures 1, 2. On D1, 46% of the total variance was explained by PC1 (29%) and PC2 (17%). On D2, 43% of the variance was explained by PC1 (25%) and PC2 (18%). On D3, 51% of the variance was explained by PC1 (31%) and PC2 (20%). On D4, 55% of the variance was explained by PC1 (29%) and PC2 (26%).

The individual farm had major influences on LI-P, especially on D4 (autumn 2012) (Figure 1), where cows from the same farm are clearly clustered together. Farm 7 cows are tightly clustered in the negative PC1 axis and positive PC2 axis, whereas Farm 14 cows are clustered in the negative PC1 and PC2 axis. The beneficial FAs n-3 and CLA9 and EPA + DPA + DHA generally occurred close together in PCA ordination space, whereas the detrimental saturated FAs C12:0, C14:0, and C16:0 are together in the opposite axes quadrants on all four sampling dates, D1–D4 (Figure 2).

Interpretation of Figure 1 is aided by cross-referencing with Figure 2 to superimpose the latter onto Figure 1. For example, on date D1, many cows from Farm 8 are associated with high levels of CLA9 and OA in milk. In contrast, Farms 2, 3, and 11 have higher saturated FA: C12:0, C14:0, and C16:0 concentrations. However, D2 cows from Farms 6, 15, and 16 are associated with the beneficial FA EPA + DPA + DHA and CLA9 and Farm 17 with n-6 and a high ratio of n-6/n-3. Across all four sampling dates, Farm 7 (yellow) stands out for producing milk with elevated n-6 content and n-6/n-3, although no farm is consistently associated with beneficial FA in milk.

### Effect of Breed on Low-Input-Production

The mean values for the components of LI-P for the eight most common breeds and crosses are shown in Table 2. Averaging data (over four dates) from multiple farms with similar breed combinations indicated that the individual parameters used to define LI-P did significantly differ between breeds, although, again, there was no difference in the stage of lactation between the breeds in this data set. The highest yielding breed was the HF (21.2 L) and the HF crosses (HFJE: 21.8 L and HFSR: 21.9 L), and HFJE had the highest fat and protein solids (1.8 kg). However, HF and the crosses had the lowest concentrations of long-chain n-3 FAs [EPA + DPA + DHA [HF: 0.20%, HFJE: 0.19%, and HFSR: 0.19%]], and HFJE and HFSR had the lowest total n-3 (both 1.3%). Additionally, HFJE had the highest concentrations of C12:0 (4%), C14:0 (11.2%), and C16:0 (32.9%). AYRX had the lowest concentration of C12:0 (3.2%), C16:0 (29.6%), and n-6/n-3 (1.0) and also had the highest concentration of OA (20.3%), CLA9 (0.99%—not significant), EPA + DPA + DHA (0.23%), and n-3 (1.7%). SH had the lowest average daily yield (17.8 L) and solids (1.5 kg), a high average cell count (261 × 10^3^ cells/ml milk), the highest concentration of n-6 (2.1%) and n-6/n-3 (1.9) and had a low concentration of EPA + DPA + DHA (0.20%), n-3 (1.40%), and CLA9 (0.74%).

There was no difference in the SCC between breeds, but 12% of SCC recordings from individual cows were above the EU standard, ranging from 400,000 to 9,000,000 cells/ml milk. This resulted in SCC having a very wide standard deviation; therefore, the median values were included in Table 2 (as well as mean values) for a more representative SCC status. The median cell counts for each breed are below 90,000 cells/ml milk. Most health treatments were given to the AYRX (0.41), whereas the NZFX (0.08) and SH (0.09) received the least.

### Low-Input-Production Score

The two LI-P scores for each breed combination are presented in Table 3. The NZFX was the highest-scoring breed, ranking first under both the weighted health and production scenarios, whereas SH was the lowest-scoring breed ranking last in both scenarios. The largest change in the LI-P score with the different weightings was HFJE, which scored fourth in the health score, but second, emphasizing production.

**Table 3:** Effect of breed on health score and production score ± standard deviation.

	**NZFX^a^**	**AYRX**	**HFJE**	**SRX**	**HFSR**	**JEX**	**HF**	**SH**	**Sig^b^**
*n*	90	100	184	140	274	121	325	80	
Health^c^ score	0.60^d^ ± 0.136	0.60 ± 0.167	0.58 ± 0.164	0.58 ± 0.143	0.57 ± 0.163	0.57 ± 0.167	0.57 ± 0.165	0.50 ± 0.133	*
Rank	1	2	4	3	5	7	6	8	
Production score	0.61 ± 0.169	0.60 ± 0.202	0.61 ± 0.194	0.59 ± 0.170	0.59 ± 0.198	0.59 ± 0.199	0.57 ± 0.198	0.50 ± 0.197	**
Rank	1	3	2	4	5	6	7	8	

a *AYRX, Ayrshire cross; HF, Holstein/Friesian; HFJE, Holstein/Friesian × Jersey; JEX, Jersey cross; NZFX, New Zealand Friesian cross; SH, Shorthorn; SRX, Scandinavian Red cross*.

b P-values < 0.05. **P < 0.01; *P < 0.05.

c *Maximum possible score = 1*.

d *Where mean values are equal the lower standard deviation dictates the rank*.

*D1, autumn 2011; D2, spring; D3, summer; D4, autumn 2012*.

## Discussion

The data collected for this paper provides valuable information from commercial farms of direct practical application for farmers, in an area lacking in the scientific literature. As a study monitoring on-farm activities, many variables are not controlled, but the statistical model mitigates some of these effects. The data collected is of sufficient quality and range to provide invaluable insights into LI-P systems in the UK. This includes the effects of breed combinations on LI-P and determining how and why breeds are suited to different farms. Although this paper does not draw definitive conclusions, it explores the current status of dairy breeding strategies and highlights how farmer's decision-making should direct future LI (cross) breeding research.

### Low-Input Production

The influence of farm management (e.g., breed, diet, calving date, and nutrition) on milk composition, yield, and animal health has been well documented (28, 29, 61, 62). These effects are seen in the PCA analyses (Figure 1), where each farm system clusters (apart from D1, with fewer records). Most organic cows were autumn calving, and many LI were spring calving (Table 1). Due to this collinearity, it would be statistically difficult to identify if management (organic vs. LI) or stage in lactation affected LI-P. Additionally, the collinearity violates the assumptions of most statistical models on the independent influence of factors; it would therefore be incorrect to separate these in an attempt to identify whether the management or lactation stage has the strongest influence on LI-P. It is clear, nevertheless, from Figure 1 that LI-P is very closely associated with individual farms. The specific aims and preferences of individual farmers result in decisions about suitable breeds for that particular system, and as these management decisions are unique to each farm, the effect of breed on LI-P is multifaceted.

### Feeding

Although the scoring system aimed to identify breeds well-suited to LI farming, there were differences in supplementary feeding between breeds, which could influence findings. The amount of concentrate feed offered was fairly consistent across breeds (from 3.0 to 4.5 kg per head per day), although conserved forage offered was more variable, ranging from 1.7 to 8.6 kg per head per day. Increasing fresh forage in the diet influences milk fat composition, raising CLA9 and omega-3 (29, 63), and if we assume fresh forage consumption is indirectly proportional to the amounts of other feeds offered (32), we could expect the ranking of the breeds to follow a similar pattern—driven by the positive influence of milk fat composition to these composite scores. However, although this holds for the best and worst ranked breeds under both scores [NZFX ranked first on both scores, had the lowest supplementary feeding, the highest concentration of CLA9 [1.03%], and the second highest concentration of n-3 [1.5%] and SH, eighth on both scores, had the highest level of supplementation offered], the ranking of all other breeds does not follow combined supplementary feeding rates. The AYRX outranked both SRX and JEX in health and production scores, whereas HFJE outranked JEX in both scores and SRX in production score; yet, both AYRX and HFJE received more supplementary feed than JEX and SRX. Despite receiving higher levels of supplementary feed than JEX and SRX, milk from AYRX cows had the highest concentration of n-3 (1.7%) and second-highest CLA9 (0.99%) among all the breeds. At the other end of the health and production ranking, JEX cows were judged seventh and sixth yet were offered the second-lowest level of supplementary feeding, hence expected to have a relatively high grazing intake. Despite the evidence that feeds management has the greatest impact on the FA profile (29, 61), this study sampled milk from a wide variety of farms and breeds where the effect of diet was possibly minimized, potentially displaying differences between the breed.

### Animal Health

SCC is an indication of udder health, cow welfare, and milk quality. Generally, if SCC is below 100,000 cells/ml milk, the cow is considered healthy, whereas above 200,000 cells/ml milk, the cow is likely to have at least one mastitic quarter, and, although some cows naturally have higher SCC, above 400,000 cells/ml milk is deemed unfit for human consumption by the EU (64). During the study, only 19% of high SCC (>400,000 cells/ml milk) cows received a health treatment (veterinary or other). Under EU organic guidelines, cows are expected to resist infection through effective management (65), suggesting that the farmers in this study were more likely to allow cows to build immunity to fight infection rather than treat with antibiotics. Interestingly, HF and HF crosses were responsible for 41% of the high cell counts, whereas only 4% of cows with SCC over 400,000 cells/ml milk were the best performing breed (NZFX), providing evidence that NZ genetics have effective health traits. Additionally, this portion of animals with high cell counts highlights the need and potential benefit of breeding for improved health traits, especially in organic production systems, when prophylactic treatment is not an option. A recent report found antibiotic use in livestock decreased 40% from 2013 to 2017 (66), but there is still pressure on dairy industries to reduce antibiotic use due to antimicrobial resistance, which already impacts human and animal health (67).

### Breeding Objectives

The effect of forage diets on milk FA profile has been well-researched (29, 46, 61), but forage conversion by diverse breeds in LI systems has not. Most of the research into forage conversion has predominantly focused on HF (68, 69). Other studies have suggested that the JE × HF cross is better suited to a pasture-based system (70–72) but only compared with HF. As a generalization, HFs were bred for their production traits rather than milk composition or health traits (73). This was reflected in this study, as cows with HF genetics had the highest yield (21.2–21.9 L/day), and HFJE had the most protein and fat solids (1.8 kg/day), but SCC was highest for HFSR (294,000 cells/ml milk) and HFJE (third highest: 248,000 cells/ml milk). Although HFs are important in the UK, and their crosses have worked well in some grazing based systems, further research into forage conversion in more diverse breeds is needed to improve LI and organic dairy systems. Although cattle diets might be the dominant factor controlling milk FA profiles, there is also evidence that heritability affects milk fat composition both within and between breeds (20, 34). This suggests a combination of feeding forage and selective breeding may optimize FA composition for consumer health. Despite breeding bodies and milk purchasers prioritizing milk fat and protein content, there is currently no premium to reward fat composition in the UK. Organic Valley's “Grassmilk™” (USA) receives a 15% premium above organic prices for n-3, CLA9 content, and n-6/n-3 ratio (74). This demonstrates a market for optimizing milk fat composition and thus creates a marketing opportunity for UK milk.

An alternative benchmark for LI dairy is the New Zealand National Breeding Objectives, in which grazing is emphasized and priority placed on forage conversion, the yield of milk components (protein and fat %), health, and fertility (75). Based on the importance of forage in NZ dairying, it is unsurprising that the NZ Friesian cross outperformed all other breeds in this study, ranking first in both performance scores (Table 3). Although the breed is an important component of management, diet is the strongest factor that influences FA composition in milk (46), whereas high intakes of forage in the diet increase milk n-3 concentrations and reduce n-6/n-3 ratio (28, 29). The contribution of milk FA profile to the LI-P score identifies a breed's ability to graze and use grass efficiently; therefore, the concentrations of n-3, CLA9, or n-6/n-3 ratio in milk could be used to predict how well forage is converted to milk.

### Effect of Breed on Low-Input-Production

The results of this study confirm that although management on individual farms affects LI-P, the breed also plays an important role. Despite ranking last under both scenarios (Table 3), shorthorns are well known for their positive temperament, high fertility, and efficiency in converting forage to milk (76), which are all metrics important for LI dairying although not formally analyzed in this study. In terms of desirable milk–fat composition, AYRX had the most desirable FA profile. However, AYRX yielded less milk (20.2 L/day) than the more productive HF crosses (21.2–21.9 L/day) and came fourth for SCC (243,000 cells/ml milk). Despite this, the AYRX ranked second in health and third in the production score. Ayrshires are commonly used in organic systems because of their ease of management, forage to milk conversion, and overall health and longevity (77). The Jersey crosses did not rank well (rank = seventh weighted health and sixth in production score), but the Jersey has many desirable traits for organic and LI systems (78). The Ayrshire, Shorthorn, and Jersey have merits beyond the scope of this study to measure; additionally, the low UK population of these breeds offers less scope for selection than the more popular HF.

Scandinavian Reds have a reputation for good udder health (79), and the SRX had the lowest average SCC (170,000 cells/ml milk); however, the HFSR had the highest average SCC (294,000 cells/ml milk), but interestingly, the median of both SR crosses was the same (73,000 cells/ml milk). This suggests that farms with a high mastitis challenge might cross HF with SR due to their reputation and breeding history, potentially instead of changing management to reduce infection risk.

The breeds in this study are generally popular and well-suited to organic and LI farming. Despite this, many of the desirable traits for organic and LI dairying were not measured in this study (forage conversion, fertility, temperament, ease of calving, etc.). It is easy to pick and choose the characteristics that could make a breed look “better” or “worse;” it can be subjective, but farmers make their decisions based on their priorities and what works best for their specific system, and despite the low score for LI-P, many of these breeds are all essential for LI and organic dairying.

### Heterosis

Another important factor to consider in a crossbreeding program is heterosis and the effects of back-crossing, as demonstrated from the breeding approach used on these organic and LI farms. All farms had at least three core breeds (Table 1), most of which get crossed and back-crossed. In this study, of the 1,070 cows selected from 17 farms, 40% were F1 (first generation crosses), 40% were F2 or subsequent generations, and only 20% were purebred. This confirms that in these LI and organic systems, cross-breeding is essential to develop robust, productive cows. As discussed, much of the published research is centered on HF crosses, which, as demonstrated by this study, are not representative of LI and organic management practices on the UK farms studied. Additionally, maximizing the benefits of hybrid vigor can be complicated and unpredictable, but challenging organic conditions often make heterosis worthwhile (80). Partially due to the emphasis on specific breeds, such as HF, there is little readily available, independent advice for farmers with alternative breeds, regarding heterosis. Further studies are needed using a diverse range of breeds to fully understand this effect and the benefits it offers (81), but as demonstrated by the predominance of crossbreeding in this study, the industry is ahead of the science—farmers are investigating the effects for themselves.

### Genotype by Environment Interaction

The genotype by environment interaction (GxE) is key to distinguishing between intensive and LI or organic breeding programs. Nauta et al. (18) first explored the GxE differences between organic and conventional dairying and reported heritabilities of SCC and production traits that warrant a re-ranking of dairy bulls for organic systems. The abundance of cross-breeding in this study indicates that farmers are learning about how (cross) breeds interact with their environments, potentially observing heterosis and GxE independently, suggesting that for LI and organic breeding objectives to be successful, the science will have to align with farming practices. Rodriguez-Bermudez et al. (23) conclude that by breeding for intensive systems, organic cows will not meet their potential due to the impact of GxE interactions on performance. To improve efficiency in LI/organic dairying, genotypes must be well-adapted to their systems, which has less emphasis on production but a greater focus on fertility and resilience (81). Keeping the GxE interaction in mind when developing and evaluating breeding programs is essential to allow livestock to meet their potential—regardless of the system that they are kept in.

To conclude, this paper highlights weaknesses in current UK breeding programs for LI and organic dairying due to limited past research on forage conversion to healthy milk and a bias toward HFs. The lack of robust scientific evidence necessary to advance breeding systems has resulted in the science-base often being behind best farming practices. Evidence from this study indicates that New Zealand Friesian and Ayrshire genetics could suit some LI/organic farms. Thorough further research is needed to explore the GxE and forage intake and conversion to meet the true potential of cows under these management systems. The ideal scenario would be for farmers to access an interactive flow chart to guide them through breed selection based on inputs, constraints, and priorities within their system, resulting in an indexing system unique to each farm.

## Data Availability Statement

The raw data supporting the conclusions of this article will be made available by the authors, without undue reservation.

## Ethics Statement

The animal study was reviewed and approved by all procedures were acceptable to internal ethical review, in accordance with EU Directive 2010/63/EU for animal experiments and approved by the Animal Welfare and Ethical Review Body at Newcastle University. Written informed consent was obtained from the owners for the participation of their animals in this study.

## Author Contributions

HD conducted the data analysis, writing, and formatting of the manuscript. EC and SS collected the data and managed all the laboratory work. RS provided statistical advice and editing. CL, GB, and SS developed the experimental design, editing, style, and formatting. All authors contributed to manuscript revision, read, and approved the submitted version.

## Conflict of Interest

The authors declare that the research was conducted in the absence of any commercial or financial relationships that could be construed as a potential conflict of interest.
