# Supplementary material for: Meeting Breeding Potential in Organic and Low-Input Dairy Farming
Source: Front Vet Sci. 2020 Oct 28;7:544149. doi: 10.3389/fvets.2020.544149 (PMC7655643; doi:10.3389/fvets.2020.544149)
Supplement: Supplementary file 1 [file Data_Sheet_1.docx]

Table 1 The average concentrate feed and conserved forage (kg DM/cow/day) fed during each season.

| Farm | Management | Concentrate (kg DM) | | | | Conserved forage (kg DM) | | | |
| --- | --- | --- | --- | --- | --- | --- | --- | --- | --- |
|  |  | D1 | D2 | D3 | D4 | D1 | D2 | D3 | D4 |
| 1 | Organic | 1.8 | 1.8 | 0.9 | 1.8 | 0.8 | 0.0 | 0.0 | 0.0 |
| 2 | Organic | 4.4 | 1.7 | 2.1 | 5.0 | 11.9 | 0.0 | 0.1 | 15.5 |
| 3 | Low-Input | 5.0 | 3.6 | 1.8 | 3.0 | 6.0 | 0.0 | 0.0 | 5.0 |
| 4 | Low-Input | 10.1 | 1.8 | 1.3 | 3.6 | 7.1 | 0.0 | 0.0 | 6.0 |
| 5 | Organic | 7.9 | 2.8 | 1.6 | 2.0 | 9.4 | 5.2 | 1.2 | 6.3 |
| 6 | Low-Input | 4.4 | 1.8 | 1.8 | 3.5 | 0.0 | 0.0 | 0.0 | 3.5 |
| 7 | Organic | 4.3 | 4.3 | 2.9 | 2.8 | 10.5 | 9.3 | 7.0 | 12.9 |
| 8 | Low-Input | 8.6 | 5.0 | 3.5 | 4.3 | 11.8 | 4.6 | 1.3 | 11.4 |
| 9 | Low-Input | 5.3 | 4.0 | 0.6 | 6.0 | 12.0 | 0.0 | 0.0 | 14.0 |
| 10 | Organic | 8.5 | 7.4 | 0.4 | 8.1 | 11.8 | 15.1 | 0.0 | 14.9 |
| 11 | Low-Input | 5.4 | 5.5 | 2.6 | 1.8 | 10.4 | 2.0 | 4.9 | 10.6 |
| 12 | Low-Input | 6.2 | 5.3 | 5.3 | 3.1 | 12.0 | 2.4 | 2.5 | 8.0 |
| 13 | Low-Input | 3.8 | 3.8 | 3.3 | 4.6 | 6.0 | 3.0 | 0.0 | 10.7 |
| 14 | Low-Input | 2.0 | 0.0 | 0.0 | 3.0 | 1.0 | 0.5 | 0.0 | 3.0 |
| 15 | Organic | 5.2 | 1.5 | 1.0 | 4.8 | 16.4 | 7.0 | 0.0 | 11.9 |
| 16 | Low-Input | 1.8 | 3.5 | 2.6 | 2.6 | 2.5 | 0.0 | 0.0 | 5.0 |
| 17 | Organic | 6.8 | 2.6 | 1.8 | 6.7 | 13.0 | 7.0 | 0.0 | 13.0 |
|  | Mean Low-Input | 5.2 | 3.4 | 2.3 | 3.6 | 6.9 | 1.2 | 0.9 | 7.7 |
|  | Mean Organic | 5.5 | 3.1 | 1.5 | 4.5 | 10.5 | 6.2 | 1.2 | 10.6 |

D1= Autumn 2011, D2=Spring, D3=Summer, D4= Autumn 2012

Table 2 Access to pasture during the day and/or night on each farm and sampling date.

|  |  | D1 | | D2 | | D3 | | D4 | |
| --- | --- | --- | --- | --- | --- | --- | --- | --- | --- |
| Farm | Management | Day | Night | Day | Night | Day | Night | Day | Night |
| 1 | Organic | Y | Y | Y | Y | Y | Y | Y | Y |
| 2 | Organic | Y | N | Y | N | Y | Y | N | N |
| 3 | Conventional | Y | Y | Y | Y | Y | Y | Y | Y |
| 4 | Conventional | Y | Y | Y | Y | Y | Y | Y | N |
| 5 | Organic | Y | Y | Y | Y | Y | Y | Y | Y |
| 6 | Conventional | Y | N | Y | Y | Y | Y | Y | N |
| 7 | Organic | N | N | N | N | Y | Y | N | N |
| 8 | Conventional | Y | N | Y | Y | Y | Y | Y | N |
| 9 | Conventional | Y | N | Y | Y | Y | Y | N | N |
| 10 | Organic | Y | N | Y | Y | Y | Y | N | N |
| 11 | Conventional | Y | Y | Y | Y | Y | Y | Y | N |
| 12 | Conventional | Y | Y | Y | Y | Y | Y | Y | Y |
| 13 | Conventional | N | N | Y | N | Y | Y | N | N |
| 14 | Conventional | Y | Y | Y | Y | Y | Y | Y | Y |
| 15 | Organic | N | N | Y | N | Y | Y | N | N |
| 16 | Conventional | Y | Y | Y | Y | Y | Y | Y | Y |
| 17 | Organic | N | N | Y | Y | Y | Y | N | N |

D1= Autumn 2011, D2=Spring, D3=Summer, D4= Autumn 2012, Y= Yes, N=No

**Table 3 Number of cows represented by each breed on each farm on all sampling dates**

| Farm | Management | Breed ^a^ | D1 ^b^ | D2 | D3 | D4 | Total |
| --- | --- | --- | --- | --- | --- | --- | --- |
| 2 | Organic | HFJE | 22 | 18 | 20 | 16 | 76 |
| 2 | Organic | SRX |  | 8 | 7 |  | 15 |
| 3 | Low-Input | HF | 15 | 20 | 9 | 18 | 62 |
| 3 | Low-Input | HFJE | 8 | 9 |  |  | 17 |
| 3 | Low-Input | HFSR |  | 12 | 7 | 10 | 29 |
| 4 | Low-Input | JEX | 8 |  |  |  | 8 |
| 4 | Low-Input | NZFX | 14 | 13 | 14 | 9 | 50 |
| 5 | Organic | HFSR | 8 |  | 8 | 8 | 24 |
| 5 | Organic | SRX | 8 | 8 | 8 |  | 24 |
| 6 | Low-Input | HFSR | 7 | 10 | 12 | 12 | 41 |
| 6 | Low-Input | SRX | 9 | 8 | 10 | 9 | 36 |
| 7 | Organic | HF |  | 20 | 15 | 12 | 47 |
| 7 | Organic | SH | 12 | 16 | 14 | 13 | 55 |
| 8 | Low-Input | HF |  | 12 | 13 | 7 | 32 |
| 8 | Low-Input | HFSR | 15 | 24 | 21 | 11 | 71 |
| 8 | Low-Input | SRX | 11 | 10 |  | 7 | 28 |
| 9 | Low-Input | HF |  |  | 13 | 7 | 20 |
| 9 | Low-Input | HFJE |  |  | 11 | 10 | 21 |
| 9 | Low-Input | NZFX |  |  | 9 |  | 9 |
| 9 | Low-Input | SH |  |  | 9 | 8 | 17 |
| 10 | Organic | HF |  | 15 | 14 | 13 | 42 |
| 10 | Organic | HFSR | 15 | 20 | 17 | 14 | 66 |
| 11 | Low-Input | HF |  |  |  | 9 | 9 |
| 11 | Low-Input | HFJE | 19 | 15 | 20 | 16 | 70 |
| 11 | Low-Input | JEX | 10 | 7 | 7 | 8 | 32 |
| 13 | Low-Input | HFSR |  | 16 | 16 | 12 | 44 |
| 14 | Low-Input | HF |  | 9 | 9 | 7 | 25 |
| 14 | Low-Input | JEX |  | 12 | 12 | 10 | 34 |
| 14 | Low-Input | SRX |  | 13 | 13 | 11 | 37 |
| 15 | Organic | AYRX |  | 9 | 8 | 8 | 25 |
| 16 | Low-Input | AYRX |  | 18 | 18 |  | 36 |
| 16 | Low-Input | JEX |  | 18 | 19 | 10 | 47 |
| 16 | Low-Input | NZFX |  | 16 | 15 |  | 31 |
| 17 | Organic | HF |  | 19 | 12 | 15 | 46 |
| 17 | Organic | AYRX |  | 14 | 16 | 9 | 39 |
| 17 | Organic | HF |  | 18 | 14 | 12 | 44 |
| 17 | Organic | SH |  |  | 8 |  | 8 |

^a^ AYRX= Ayrshire cross, HF=Holstein/Friesian, HFJE= Holstein/Friesian x Jersey, JEX= Jersey cross, NZFX= New Zealand Friesian cross, SH= Shorthorn, SRX= Scandinavian Red cross.

C12:0= Lauric Acid, C14:0= Myristic Acid, C16:0= Palmitic Acid, CLA.9=Conjugated linoleic acid (C18:2, c9t11 isomer), n3=omega-3, n6=omega-6, n6n3= omega-6/ omega-3 ratio, EPA+DPA+DHA= EPA=Eicosapentaenoic Acid + DPA=Docosapentaenoic Acid + DHA=Docosahexaenoic Acid, SCC= Somatic Cell Count, Treatments= Health Treatments
